# COVID-19 Vaccination in Patients with Severe Asthma on Biologic Treatment: Safety, Tolerability, and Impact on Disease Control

**DOI:** 10.3390/vaccines9080853

**Published:** 2021-08-04

**Authors:** Marco Caminati, Gabriella Guarnieri, Veronica Batani, Elena Scarpieri, Anita Finocchiaro, Fulvia Chieco-Bianchi, Gianenrico Senna, Andrea Vianello

**Affiliations:** 1 Department of Medicine, University of Verona, 37124 Verona, Italy; veronica.batani@univr.it (V.B.); elena.scarpieri@univr.it (E.S.); gianenrico.senna_01@univr.it (G.S.); 2 Respiratory Pathophysiology Division, Department of Cardiac, Thoracic, Vascular Sciences and Public Health, University of Padova, 35121 Padova, Italy; gabriella.guarnieri@unipd.it (G.G.); fulvia.chiecobianchi@aopd.veneto.it (F.C.-B.); andrea.vianello.1@unipd.it (A.V.); 3 Asthma Center and Allergy Unit, University of Verona and Verona University Hospital, 37124 Verona, Italy; anita.finocchiaro@aovr.veneto.it

**Keywords:** Pfizer mRNA SARS-CoV-2/COVID 19 vaccine, severe asthma, biologics, safety, tolerability, quality of life

## Abstract

Background: COVID-19 vaccination has been recommended for severe asthmatics. We aimed to evaluate the safety, tolerability, and impact on disease control and patient’s quality of life of the mRNA SARS-CoV-2/COVID-19 vaccine in severe asthma patients regarding biologic treatment. Methods: Severe asthmatic patients regularly managed by two big allergy and respiratory referral centers were offered to undergo Pfizer COVID 19 vaccination at the hospital site. Patients filled in an adverse events questionnaire after the first and second dose, as well as the Asthma Control Test (ACT) and Asthma Quality of Life Questionnaire (AQLQ). Results: Overall, 253 patients were vaccinated; only 16 patients refused. No serious events were detected. Less than 20% of patients reported side effects, most of which were classified as very common side effects. No differences were reported according to the ongoing biologic drug. A significant improvement in both ACT and AQLQ was observed between the first and the second dose administration. Conclusions: Our data confirm the optimal safety and tolerability profile of mRNA SARS- CoV-2/COVID-19 in severe asthma patients on biologic treatment, as well as their positive attitude towards COVID-19 vaccination. The negligible proportion of patients reporting side effects and the absence of asthma exacerbations are relevant to support the COVID-19 vaccination campaign in severe asthma patients worldwide.

## 1. Introduction

The available evidence suggests that bronchial asthma cannot be considered an independent risk factor for COVID-19 [1,2,3]; however, asthmatic patients, when admitted to intensive care for COVID-19, are likely to experience a more severe disease [2]. Furthermore, patients affected by severe asthma (GINA step 4/5) and suffering from difficult to control or uncontrolled disease should be considered at higher risk of worse COVID-19 outcomes [1,3].

When talking about severe asthma, the relationship between the currently marketed monoclonal antibodies and SARS-Cov-2 infection is still under debate, although a protective role of biologic treatment had been postulated [4,5].

The recent availability of vaccines against COVID-19 has the potential to dramatically reduce the risk of the disease and the related exacerbations in patients with more severe respiratory diseases, including severe asthmatics. In fact, COVID-19 vaccination has been recommended for severe asthmatics, whether on biologic treatment or not, and under the pathophysiological perspective, no specific risk factors for adverse events have been identified for that population [4,6]. However, the recent report of severe immediate reactions after vaccine administration, as well as the unknown potential interactions with concomitant biologic therapies, may raise some concerns about its safety in fragile populations [7,8].

Real word data on the safety, tolerability, and impact on disease control of the COVID-19 vaccination in patients with severe asthma on biologic treatment are still lacking. The aim of the present study is to evaluate the safety of the mRNA SARS- CoV-2/COVID-19 vaccine in a population of severe asthmatics, most of them treated with monoclonal antibodies, as well as to assess the impact of vaccination on the control of the disease and on patient’s quality of life.

## 2. Materials and Methods

Severe asthmatic patients regularly managed by two allergy and respiratory referral centers for severe asthma located in the Northeast of Italy (Verona and Padua), whether or not on biologic treatment, were offered to receive the mRNA SARS-CoV-2/COVID-19 (Pfizer) vaccination at the hospital site. Allergy history was carefully re-assessed in order to exclude specific risk factors for vaccine related adverse reaction, or permanent/temporary contraindications according to the current recommendations [9,10]. In particular, ongoing acute asthma exacerbation and oral steroid daily dose > 10mg prednisolone or equivalent were considered vaccination exclusion criteria [11,12]. In the case of ongoing treatment with monoclonal antibodies for severe asthma, a 48-h interval between COVID-19 vaccination and the biologic drug was planned.

Patients filled in a vaccination-related adverse events questionnaire after the first and second dose (see Appendix A). In particular, patients were asked to indicate which symptoms they observed from a list of clinical manifestations, classified as follows: very common reactions, including pain and swelling at the injection site, weakness, headache, myalgia, arthralgia, and fever; common reactions, including erythema at the injection site and nausea; uncommon reactions, including lymphadenopathy, illness, and diffuse pain; and rare reactions, including facial asymmetry and severe allergic reaction. The list and classification of symptoms was based on the information provided by the manufacturer [13].

Furthermore, the Asthma Control Test (ACT) and Asthma Quality of Life Questionnaire (AQLQ) were administered at the time of first and second dose injection, as well as 35 days after.

A Wilcoxon test was used to compare the ACT and AQLQ values after the first and after the second vaccine dose. Side effects rates were compared using the exact Fisher test. A *p* value of less than 0.05 was considered significant.

## 3. Results

Overall, 273 patients were contacted. Among them, 16 patients (5.9%) refused the vaccination, while four subjects (1.5%) were excluded because of ongoing acute asthma exacerbation and/or oral steroid daily dose >10 mg prednisolone or equivalent. The vaccinations were finally administered to 253 severe asthmatic patients. Table 1 summarizes the population’s characteristics; 251 patients returned the vaccination-related adverse events questionnaire.

**Figure 1:**
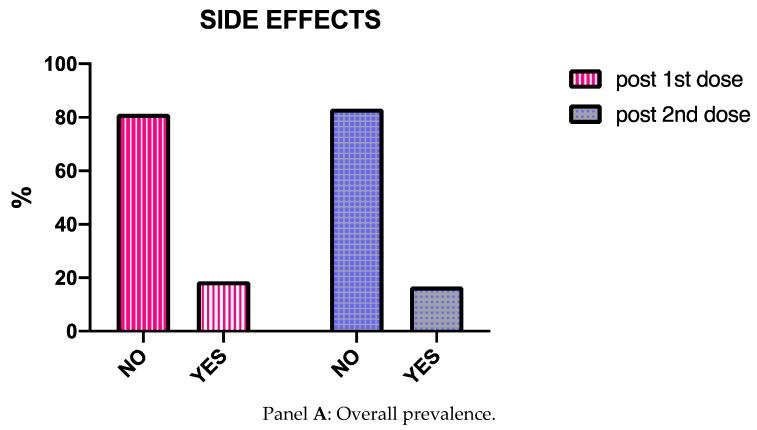

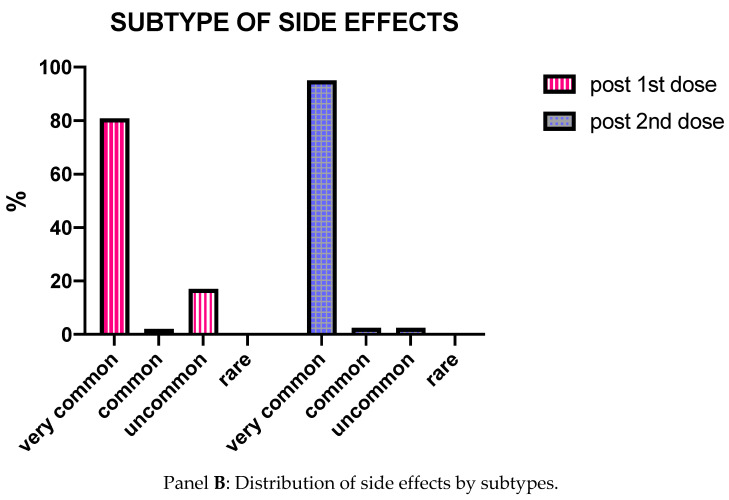
Side effects in the general population.

As highlighted in Figure 1, Panel A, 81.3% and 83.3% of patients did not report side effects following the first and second dose, respectively. When considering the patients experiencing adverse events, most of them reported very common side effects (80.8% after the first dose, and 95% after the second dose, Figure 1, Panel B).

Common side effects were described by 2.1% of patients after the first vaccine administration, and by 2.5% after the second. Following the first dose, 17.2% of patients recorded uncommon adverse events, while 2.5% reported the same side effect subtype after the second dose. No rare side effects were experienced. No premedication with antihistamine/paracetamol was recommended or referred by any patients.

**Figure 2:**
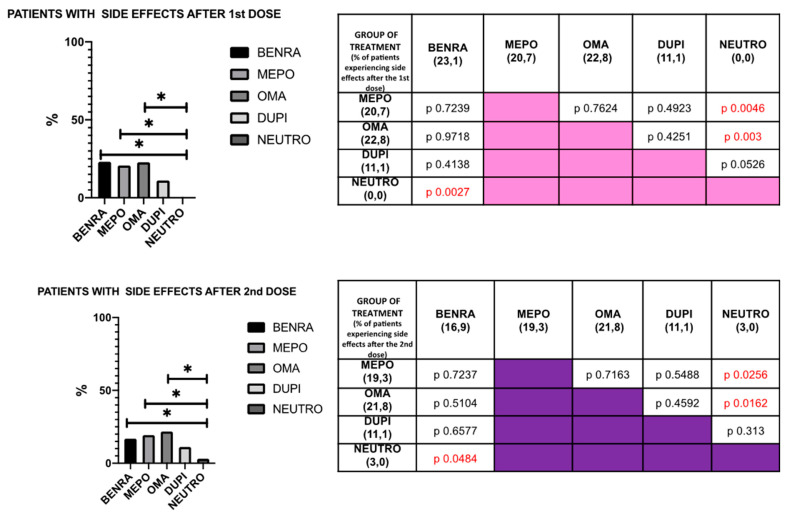
Overall distribution of the reported side effects by biologic drugs. Benra—Benralizumab; Mepo—Mepolizumab; Oma—Omalizumab; Dupi—Dupilumab; Neutro—Neutrophilic/Th2 low asthma patients not undergoing biologic treatment. * and red numbers: *p* value < 0.05.

**Figure 3:**
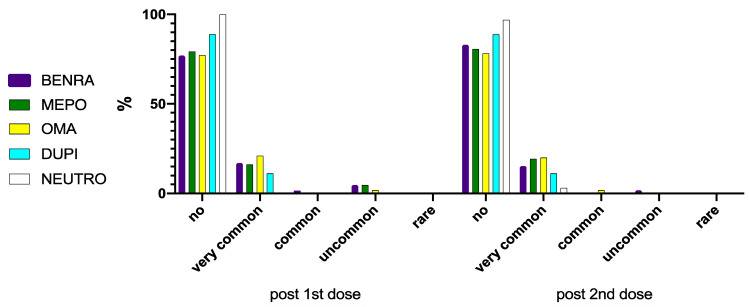
Distribution of the different side effects classes by biologic drugs. Benra—Benralizumab; Mepo—Mepolizumab; Oma—Omalizumab; Dupi—Dupilumab; Neutro—Neutrophilic/Th2 low asthma patients not undergoing biologic treatment.

When clustering the vaccinated population by biologic drugs, no significant differences could be described in terms of the reported side effects (Figure 2 and Figure 3). Overall, the proportion of asthma patients not undergoing any biologic treatment experiencing adverse events was significantly lower when compared with the treated subjects (Figure 2 and Figure 3).

**Figure 4:**
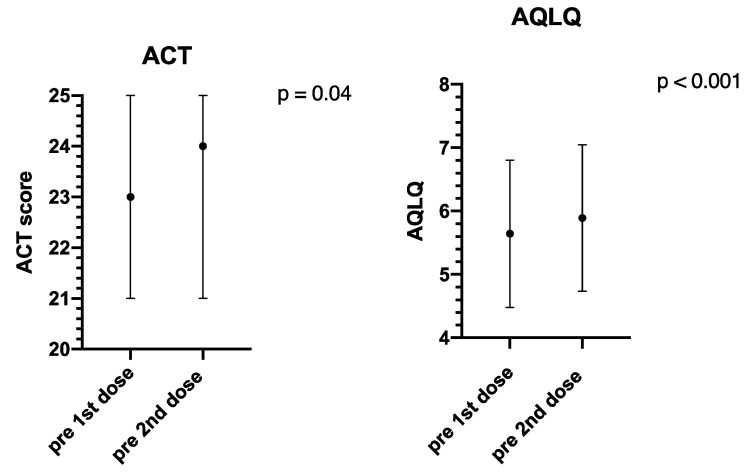
Comparison of patient reported outcomes before the first and the second doses. ACT—Asthma Control Test; AQLQ—Asthma Quality of Life Questionnaire. A *p* value of less than 0.05 is considered significant.

Patient reported outcomes are described in Figure 4. Overall, both ACT and AQLQ significantly improved from the first to the second time-point. The median ACT was 23 (IQR 21–25) at the time of the first dose administration and 24 (IQR 21–25) before the second dose. Median AQLQ increased from 5.9 (IQR 5.2–6.5) to 6.16 (IQR 5.5–6.7).

## 4. Discussion

Our real-life study investigated the safety and tolerability of mRNA SARS-CoV-2/COVID-19 vaccination in severe asthma patients, most of them undergoing biologic treatment. Its impact on asthma control was also evaluated. According to our findings, no serious adverse events were detected. Less than 20% of patients reported side effects, most of them classified as very common side effects. No differences were observed when clustering the study population a ongoing biologic drug use. Overall, the asthma patients not treated with biologics experienced less adverse events. In terms of patient reported outcomes, a significant improvement of both ACT and AQLQ was observed between the first and the second dose administration.

When considering the mechanism of action of mRNA SARS-CoV-2/COVID-19 vaccination, a higher risk of COVID-19 vaccine related adverse events in severe asthma patients in comparison with the general population cannot be hypothesized [13]. Similarly, the efficacy of the same vaccination is not limited by the ongoing anti-asthmatic biologic treatment. In fact, the cytokines commonly targeted by the currently marketed biologics are not directly involved in the antiviral response, and data from studies on other antiviral vaccines in treated severe asthma patients confirm that biologics do not hamper the immunological reactivity [14,15]. Furthermore, exploratory data suggest that anti SARS-Cov-2 antibody production does not significantly differ in severe asthma patient, whether on biologic treatment or not [16]. The data mentioned above, together with the potential higher risk of worse COVID-19 outcomes in patients affected by severe asthma [1,3], provide a robust rationale for recommending COVID-19 vaccination in that population [6]. However, up until now, no real-life data on the safety and tolerability of mRNA SARS-CoV-2/COVID-19 vaccination are available. According to our study, no serious adverse events were experienced by vaccinated severe asthma patients on biologic drugs, and less than 20% of them reported side effects, most of which were classified as very common side effects, without relevant differences between the first and the second dose. Of note, a 48-h interval between vaccine administration and biologic drug was adopted, even if no univocal recommendations are currently available. Overall, asthma patients not undergoing biologic treatment for severe asthma reported less adverse events. Differently from the other group, they were affected by neutrophilic or Th2 low asthma, not characterized by atopy or increased blood eosinophils. In a post-marketing safety surveillance of quadrivalent recombinant influenza vaccine, atopy may be associated with more frequent adverse event, but true increased susceptibility in atopic patients has not been demonstrated [17].

The other relevant point is whether COVID-19 vaccination may act as an asthma exacerbation trigger, although not expected when considering the vaccine mechanism of action [13]. In our study, the ACT values before the first and the second administration showed a significant increase, ruling out the risk of asthma exacerbations related to the COVID-19 vaccine. Of note, all of our patients were on optimal asthma control at the time of the administration, and poor asthma control was considered a temporary contraindication to the COVID-19 vaccine. On the other hand, a cause–effect relationship with the vaccination itself cannot probably explain the improvement of ACT median values between the first and the second dose, as the pharmacological plausibility of such an effect is difficult to demonstrate. ACT individual variability is well known within the severe asthma clinical profile [18], and it may more likely account for our observation, as suggested by the IQR interval stability, despite the variation of median values.

AQLQ also significantly increased from the first to the second assessment, at the time of the second dose administration. This could be related to patients’ positive attitude towards the protective role of the COVID-19 vaccination, as they are aware that severe asthma potentially increases their individual susceptibility to COVID-19 or to a more severe related condition [1,3]. According to the literature, age (old vs. young) and vaccine category (non-mRNA vs. mRNA) represent the major determinants of hesitancy towards the COVID-19 vaccination [19]. However, the data come from a general population sample, which may consistently differ in terms of attitude toward the vaccine in comparison with severe asthma patients or other selected categories.

## 5. Conclusions

To the best of our knowledge, our data provide the first real-life description of the optimal safety and tolerability profile of mRNA SARS-CoV-2/COVID-19 vaccine in severe asthma patients in biologic treatment, without relevant differences according to the specific monoclonal antibody. Our findings need to be confirmed in larger studies and thorough post-marketing surveillance protocols. However, both the negligible proportion of patients reporting side effects and the absence of asthma exacerbation episodes are relevant to support and carry on the COVID-19 vaccination campaign in severe asthma patients worldwide.

## Figures and Tables

**Table 1:** General overview of the study population.

Total, *n*	253
Female, *n* (%)	141
Male, *n* (%)	112
Age, median (IQR)	57 (49–65)
Benralizumab treatment, *n* (%)	65 (26)
Mepolizumab treatment, *n* (%)	88 (33)
Omalizumab treatment, *n* (%)	58 (25)
Dupilumab treatment, *n* (%)	9 (3)
Neutrophilic severe asthma, *n* (%)	33 (13)

IQR—inter quartile range.

## Data Availability

The data presented in this study are available from the corresponding author upon reasonable request.
